# Costs associated with adverse events from remission induction for children with Acute Lymphoblastic Leukemia (ALL)

**DOI:** 10.1186/s12913-022-08676-x

**Published:** 2022-12-14

**Authors:** Eréndira Mejía-Aranguré, Alfonso Reyes-López, Luis Enrique Juárez-Villegas, Yosef Olaf Hernández-Olivares, Alberto Daniel Saucedo-Campos, Gabriela Hernández-Pliego, Silvia Martínez-Valverde, Leticia A. Barajas-Nava, Juan Garduño-Espinosa

**Affiliations:** 1 Programa de Maestría y Doctorado en Ciencias Médicas, Odontológicas Y de La Salud, Universidad Nacional Autónoma de México, Ciudad de México, México; 2 Medicina Basada en Evidencia, Unidad de Investigación, Hospital Infantil de México Federico Gómez, Ciudad de México, México; 3 Present Address: Dr. Márquez 162, Col. Doctores. Del. Cuauhtémoc, C.P. 06720 Ciudad de México, México; 4 Centro de Estudios Económicos y Sociales en Salud, Hospital Infantil de México Federico Gómez, Ciudad de México, México; 5 Departamento de Oncología Pediátrica, Hospital Infantil de México Federico Gómez, Ciudad de México, México; 6 Dirección de Investigación, Hospital Infantil de México Federico Gómez, Ciudad de México, México

**Keywords:** Children, Cost, Adverse event, Leukemia, Chemotherapy, Toxicity, Remission

## Abstract

**Background:**

ALL is the most frequent hematological tumor in children, so during remission induction chemotherapy protocol (RICP) adverse events (AEs) may appear. The public program in Mexico in charge of financial support to oncologic children without social security delivered a fix amount for ALL chemotherapy, but additional money needed to treat any other unexpected condition should be taken from the budget of the oncologic healthcare providers. So the purpose of our study was to estimate and evaluate the direct medical costs associated to EAs during RICP in children with ALL.

**Methods:**

This study was retrospective, longitudinal, and observational based on medical records review of patients in RICP. The CTCAE was used to identify and classify AEs according to a SOC category. We focused on extracting resources data that were consumed both for inpatients and outpatients AEs. A micro-costing approach was adopted which involve quantification of each healthcare resource consumed by the hospital multiplying them by unit cost. The probability distributions of data were evaluated to identify the appropriated statistical tests to be used for comparisons between groups that were performed with Wilcoxon rank sum test. Generalized linear models (GLM) were adjusted to evaluate the effects of patient characteristics on total cost.

**Results:**

Forty patients accumulated 204 inpatient and 81 outpatient AEs during RICP. Comparison of total costs between groups showed an incremental cost of $7,460.23 likewise attributable to AEs. The total cost of a pediatric patient undergoing RICP without adverse events was $3,078.36 and the total cost of a patient with AEs exceeds it threefold.

**Conclusions:**

The costs associated with AEs during RICP in Mexican children with ALL representing a high burden for the healthcare provider. Generalized linear models showed that variables such as sex, risk category and alive status are associated with the total costs of AEs. This is the first study aiming to analyze the effect of ALL-related AEs on health care costs in pediatric population, so our results may help not only to local decision making but also it may contribute to the research agenda in this field.

**Supplementary Information:**

The online version contains supplementary material available at 10.1186/s12913-022-08676-x.

## Background

ALL is the most frequent hematological neoplastic disease reported at pediatric age. In developed countries 96% to 99% of pediatric patients achieve disease remission [1–4] with a treatment regimen similar to the currently used at the Hospital Infantil de México Federico Gómez (HIMFG). The RICP include vincristine, L-asparaginase, one anthracycline and one glucocorticoid which are all administered during 4 weeks resulting in ALL remission (less than 5% blasts per bone marrow aspirate) in a short time favoring high survival rates [1]. An AE is any unfavorable and unintended sign, symptom, or disease temporally associated with the use of a medical treatment or procedure that may or may not be considered related to the medical treatment or procedure [5]. AEs related to treatment toxicity are usually acute and not serious, but when they are serious the patients may even die. Some AEs requiring hospital treatment such as bleeding, pancreatitis, febrile neutropenia, septic shock or pancytopenia, often occur during RICP and may entail treatment interruption until the patient meets the clinical requirements necessary to complete treatment, which may involve considerable cost to the hospital.

The estimated mortality during remission induction reported for USA is 1.12% [6], however in Mexico it ranges between 7% [7] and 15% [8] in patients who achieved complete remission, whereas chemotherapy-associated mortality in children is around 51.4% in developing countries [9], being the main causes: infections, septic shock, bleeding [7–10], severe anemia, tumor lysis syndrome and hyper leukocytosis [11–15]. Some clinical characteristics of patients have been associated with the presence of AEs such as sex, risk standard or high (ALL risk category) of neoplastic relapse (more than 5% of blasts) [16] and early response to RICP treatment [17, 18]. Studies on the cost of AE in pediatric patients with ALL are scarce; for the adult population, the cost of USD 33,189 was determined for inpatient treatment of febrile neutropenia [19–21], the most frequent AE reported in patients with ALL of HIMFG [11]. A systematic review found that hospital-acquired adverse drug reactions (adverse events) translate into £380 million per year in UK while in Canada the overall cost of AE related admission to the emergency department and succeeding hospitalizations was quantified as approximately $13.6 million USD [22].

At the time the study was conducted, the public program in Mexico in charge of financial support to oncologic children without social security delivered a fix amount for ALL chemotherapy, but additional money needed to treat any other unexpected condition should be taken from the budget of the oncologic healthcare providers, therefore our research purpose was to estimate the costs of AEs associated with ALL treatment in children.

## Methods

The study design was retrospective, longitudinal, and observational; data were extracted from 53 (68 were review) medical records of patients that received treatment for remission induction of ALL from 2015 to 2019 at the Hospital Infantil de México Federico Gómez. Inclusion criteria were the following: diagnosis of ALL B-cell, standard or high risk for ALL relapse, previously (before neoplastic diagnosis) healthy (no chronic, congenital or neoplastic diseases), patients who were started on the same antineoplastic regimen and completed it, death due to complications during RICP; exclusion criteria were the following: Philadelphia chromosome ( +), T-cell ( +), diagnosis of Down syndrome. The CTCAE was used first to discriminate the patients with AEs from those without AEs, then it was used to classify the AEs according to a SOC category [5]. Hospitalization was used as a proxy of AEs severity since the medical notes not allowed us to differentiate the grades of severity according to CTCAE, so the outpatient management of AEs was assumed as a low grade severity of them, which in turn not favored the costing of AEs according to severity. We focused on extracting resources data that were consumed both for inpatients and outpatients AEs during the RICP. Synonyms were used for those AEs extracted from the clinical notes not matching CTCAE names and none were dropped. Data about medical resources included length of stay, surgical and specialized procedures, laboratory and radiology test, blood products, general medications, inpatient and outpatient medical consultations and chemotherapy treatment (RICP). Costs estimations were aggregated by type of medical care (inpatient and outpatient). The study protocol was approved by the research committee of the HIMFG with the number HIM-DIC-SR-2020–004, and it was performed in accordance with principles of good research practice. Since the retrospective nature of our study no written or verbal consent was necessary.

Cost estimations were done from the perspective of the health care provider (HIMFG) which is a tertiary referral hospital located in Mexico City which belongs to the Health National Institutes, therefore the focus was on direct medical costs. A micro-costing approach was adopted which involve quantification of each healthcare resource consumed by the hospital during RICP for inpatient and outpatient patients care and multiplying it by its unit cost; a time horizon lower than one year (90 days) was utilized so no costs discounting was necessary. Information about prices and unit costs of medical resources were taken from the hospital tariff payment system. Body weight or body surface area information was used for calculation of total cost of pharmacotherapy. All cost estimations were expressed in US dollars of 2020.

Descriptive statistics included calculation of central tendency and dispersion measures for continuous variables, while categorical variables were analyzed with relative frequency tables. The probability distributions of data were evaluated to identify the appropriated statistical tests to be used (parametric or non-parametric tests) for comparisons between groups.

We collected clinical data such as risk category (standard or high), age, weight, height, sex, death, time to ALL remission, length of stay, number of hospitalizations. Generalized linear models (GLM) were adjusted to evaluate the effects of patient characteristics on total cost, because they allow dealing with biased data and directly modeling heteroscedasticity, giving the possibility of having a specification that approximates the real process of generating economic data in the health sector. Therefore, to choice the proper link function and variance distribution family of the GLM, the specification tests were first performed with Akaike (AIC) and Bayesian (BIC) information criteria whose lowest values help for this purpose. We also estimated the variance inflation factor (VIF) to verify collinearity between covariates. All statistical procedures were performed with the version 16 of STATA program.

## Results

**Fig. 1:**
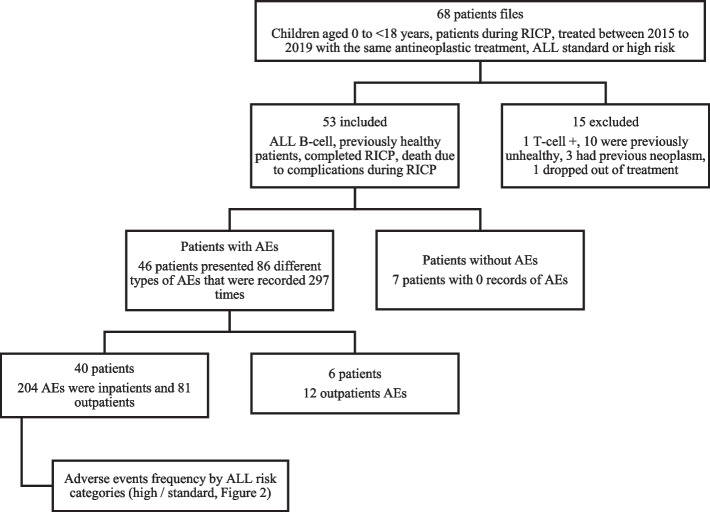
Patient selection flowchart

The clinical characteristics of patients are shown in Table 1. Fifty- three medical records met inclusion criteria for review, of which 30 were high-risk category and 23 standard risk ALL. No adverse events were found in 7 patients, so this group was used as a reference for comparison purposes (Fig. 1).

**Table 1:** Characteristics of patients with and without AEs

Variables	Patients with AEs (*n* = 46)	Patients without AEs (*n* = 7)	*p* value
**Age (years)**	7.00 (4.44)	6.00 (2.64)	0.802^a^
**Weight (kg)**	27.04 (18.17)	22.40 (11.46)	0.813^a^
**Height (cm)**	115.66 (26.68)	111.00 (16.27)	0.727^a^
**Female, n (%)**	25 (54.35)	3 (42.86)	0.694^a^
**High risk ALL, n (%)**	27 (58.70)	3 (42.86)	0.451^a^
**Standard risk ALL, n (%)**	19 (41.30)	4 (57.14)	0.451^a^
**Completed RICP, n (%)**	43 (93.48)	7 (100)	0.487^a^
**ALL remission, n (%)**	45 (97.83)	7 (100)	0.694^a^
**Deaths, n (%)**	4 (8.70)	0 (0)	0.417^a^
**Time to get ALL remission (days)**	15.55 (5.97)	15.28 (3.35)	0.747^a^
**First hospital length of stay (days)**	9.33 (13.65)	1.86 (4.91)	0.006^a^
**Second hospital length of stay (days)**	6.62 (9.74)	0.14 (0.37)	0.101^a^
**Third hospital length of stay (days)**	1.35 (6.25)	0 (0)	0.491^a^
**Amount of AEs by patient**	6.46 (6.06)	0 (0)	< (0) 01^a^

The values shown are mean and standard deviations except for percentages

*AEs* Adverse events

^a^Wilcoxon rank sum test

### Adverse events

**Fig. 2:**
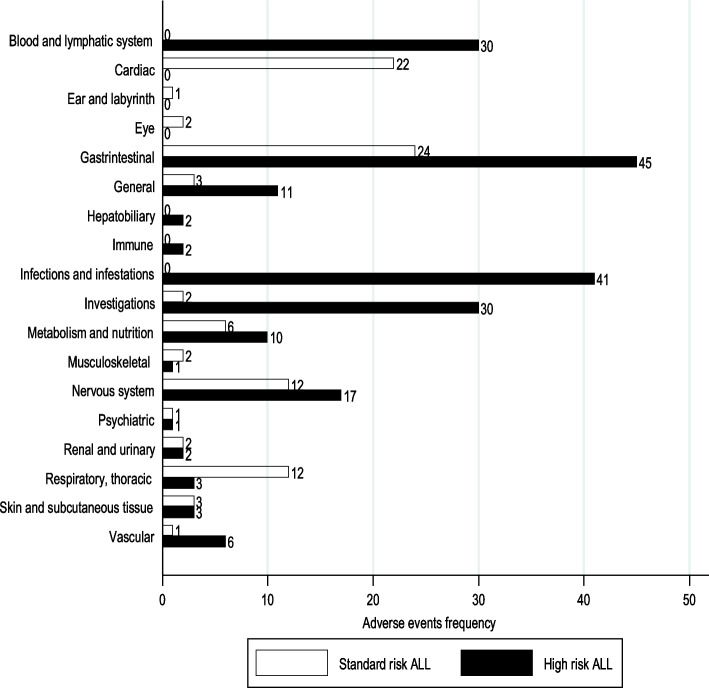
Adverse events frequency by ALL standard and high risk of neoplastic relapse

Eighty-six different conditions were located in the medical records, 82 were identical to a CTCAE terms and 4 were synonyms; these terms were found with repetition in 297 occasions and grouped to the corresponding SOC categories (Fig. 2). Forty patients (75.5%) accounted for by 204 inpatients and 81 outpatients AEs, 6 patients (11.3%) only experienced 12 outpatients AEs and 7 patients (13.2%) did not suffer AEs (Table 1). Only 13 patients completed the RICP within 4 weeks, while all inpatients exceeded 4 weeks. The mean number of AEs per patient was 6.5 (median: 4, SD: 6.1) and the frequency of inpatient AEs in RICP exceeds that of outpatient by three to one. The SOC category gastrointestinal diseases (Fig. 2) accumulated the highest number (69) of AEs, the most frequent were abdominal pain 13, neutropenic colitis 11, mucositis 9 and pancreatitis 8. We can see that patients diagnosed with high risk ALL presented the highest number of AEs. It is remarkable that they presented almost twice as many gastrointestinal AEs and were the only ones who presented infections/infestations and blood and lymphatic system AEs. The 41.3% of patients with AEs were standard risk.

A wide variety of clinically important AEs were also found, which were classified into the following SOC categories: cardiac, hepatic, immunological, skeletal muscle, nervous system, psychiatric, respiratory, thoracic, mediastinal, vascular, and urinary system. Febrile neutropenia was the most frequent AE in the sample (25 events) followed by tachycardia (21) and sepsis (15).

All patients with AEs suffered by more than one event so the mix of different conditions made us difficult the identification of data about resources devoted specifically for each AE management.

**Fig. 3:**
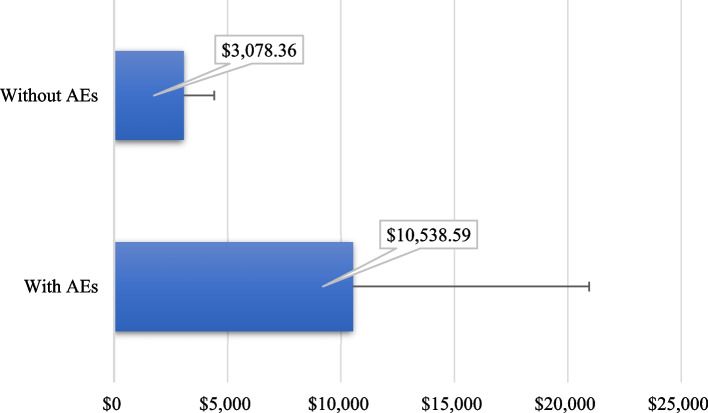
Comparison of the total cost of care for children with and without AEs

The Table 2 shows the costs results for inpatient care group (*n* = 39) with AEs highlighting the great dispersion of cost. The main cost driver for this group was general medications followed by length of stay. Since only one patient in the group without AEs experienced hospitalization (started before RICP) the corresponding costs results were not included in Table 2, but all they were low compared with the mean cost of the group with AEs ($1,799 difference for general medications, $837 for laboratory tests and around $480 for blood products and length of stay, respectively). It is important to emphasize that the inpatient management of AEs resulted in the interruption of RICP until hospital discharge, therefore no chemotherapy cost was included as part of hospital costs, but for the subsample (*n* = 39) that were hospitalized due to AEs the mean cost of chemotherapy was $4,265.35 (SD: $2,117.51) which was funded by the hospital anyway. It was not so for the group with outpatient care, especially in the group with AEs (*n* = 7) since chemotherapy was not interrupted, therefore the corresponding cost was taken into account (Table 3); for this group chemotherapy cost was prominent although it was twofold for outpatient AEs maybe due to modifications in chemotherapy decided by oncologist. Comparison of total costs between groups (with and without AEs) showed an incremental cost of $7,460.23 likewise attributable to adverse events (Fig. 3).

**Table 2:** Estimations of inpatient care costs for children with adverse events during RICP

Medical resources	Mean	SD	Median
**Length of stay**	$1,558.73	$1,362.94	$1,078.67
**Surgery**	$126.24	$378.61	$0.00
**Special procedure**	$280.53	$946.49	$0.00
**Laboratory test**	$836.76	$1,924.00	$396.42
**Diagnostic radiology test**	$36.10	$75.13	$0.00
**Blood products**	$479.51	$1,272.79	$27.12
**Inpatient medical consultation**	$755.93	$938.69	$415.82
**General medications**	$3,095.64	$5,939.04	$901.74
**Total inpatient**	$7,169.45	$11,117.75	$2,964.22

*SD* Standard deviation

**Table 3:** Estimations of outpatient care costs for children with and without adverse events during RICP

Medical resources	Patients with AEs (*n* = 7)	Patients without AEs (*n* = 6)	Mean difference	*p*-value
Laboratory test, mean (SD)	$23.13 ($61.20)	$14.55 ($35.65)	$8.58	1.000^a^
Blood products, mean (SD)	$3.87 ($10.25)	$0.00 ($0.00)	$3.87	0.354^a^
Outpatient medical consultation, mean (SD)	$82.03 ($24.06)	$69.30 ($41.06)	$12.73	0.422^a^
Chemotherapy (RICP), mean (SD)	$4,893.91 ($1,630.34)	$2,788.57 ($1,315.28)	$2,105.34	0.032^a^
Total, mean (SD)	$5,002.95 ($1,589.53)	$2,872.42 ($1,340.27)	$2,130.53	0.032^a^

*SD* Standard deviation, *AEs* Adverse events

^a^Wilcoxon rank-sum test

**Fig. 4:**
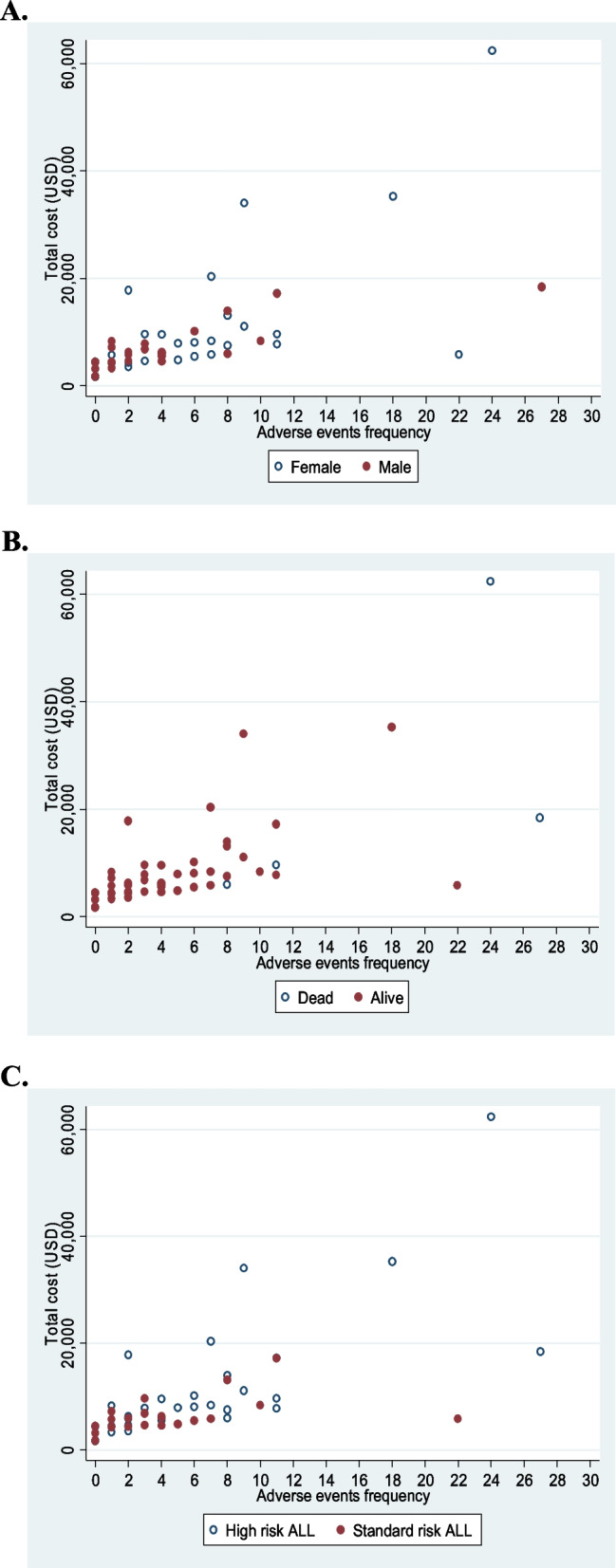
**A** Correlation between number of AEs and total cost of care by sex. **B** Correlation between number of AEs and total cost of care by mortality. **C** Correlation between number of AEs and total cost of care by ALL risk category

The correlations between total cost and the amount of AEs supported the results of Tables 2 and 3, since the scatter plots showed increasing trends by sex, alive status, and ALL risk category; Fig. 4A shows that girls with AEs had a higher cost, as well as dead patients (Fig. 4B) and finally, patients at high risk of relapse had more AEs and total cost (Fig. 4C).

### GLM models

The Table 4 shows the results of the specification tests performed by means of the Akaike and Bayesian information criteria, denoting that the GLMs with the lowest values were those with gamma family and log-link function as well as square root link function; having adjusted the logarithmic and square root link functions, together with the Gaussian, Poisson and gamma variance distribution families, i.e. six different models were evaluated for which comparisons of the likelihood (log) values were performed. Therefore, we decided to use the log-link function for GLM. We estimated two GLMs (Table 5) to know the effect of AEs as a binary predictor as well as a discrete covariate. The GLM 1 estimated an incremental cost of AEs near $4,000 which suggests that the calculated total cost difference (USD $7,460.23) may not be fully attributable to AEs occurrence, while for each additional adverse event the total cost increase $565 as we can see in the GLM 2. It is worth noting that the greatest effect was observed for death which suggests that cost of AEs increase as patients die from adverse events. Generalized linear models show that variables such as sex, age, risk of neoplastic relapse and death/ alive are associated with the presence, frequency and cost of AE. We also determined the variance inflation factor (VIF) test to verify collinearity and observed in the Table 6 none of the VIFs exceed the value 10 which is indicative of the presence of collinearity.

**Table 4:** Results of specification tests based on Akaike and Bayesian information criteria

GLM Model	AIC	BIC
Gamma family log-link function	1,079.45	1,081.42
Gamma family square root link function	1,079.45	1,081.42
Gaussian family log-link function	1,128.05	1,130.02
Gaussian family square root link function	1,128.05	1,130.02
Poisson family log-link function	346,506.70	346,508.70
Poisson family square root link function	346,506.70	346,508.70

*GLM* Generalized linear models, *AIC* Akaike, *BIC* Bayesian

**Table 5:** Results of GLMs to assess the effects of patient characteristics on the total cost of AEs

Covariates	**Coefficients**	**Estimates (USD)**	***p*-value**	**95% confidence intervals (USD)**	
**GLM 1**					
** Adverse events (0 = absent, 1 = present)**	0.467	$3,750.06	0.013^a^	$802.89	$6,697.22
** Sex (0 = male, 1 = female)**	0.160	$1,289.68	0.263^a^	-$966.69	$3,546.06
** Age (years)**	0.002	$12.95	0.938^a^	-$313.52	$339.43
** Risk of neoplastic relapse (0 = standard, 1 = high)**	0.302	$2,430.59	0.043^a^	$80.94	$4,780.24
** Time to get ALL remission (days)**	0.041	$330.50	0.004^a^	$105.31	$555.69
** Hospitalization (0 = no, 1 = yes)**	0.448	$3,601.37	0.002^a^	$1,302.69	$5,900.04
** Deaths**	0.780	$6,273.87	0.028^a^	$693.88	$11,853.87
** Intercept**	6.859		0.000^a^		
**GLM 2**					
** Adverse events (count predictor)**	0.072	$564.64	0.002^a^	$202.07	$927.22
** Sex (0 = male, 1 = female)**	0.042	$330.42	0.717^a^	-$1,453.15	$2,113.99
** Age (years)**	0.005	$40.83	0.746^a^	-$206.29	$287.95
** Risk of neoplastic relapse (0 = standard, 1 = high)**	0.259	$2,020.45	0.048^a^	$18.96	$4,021.94
** Hospitalization (0 = no, 1 = yes)**	0.303	$2,364.41	0.028^a^	$250.26	$4,478.56
** Time to get ALL remission (days)**	0.029	$226.73	0.002^a^	$80.36	$373.11
** Deaths**	-0.169	-$1,320.33	0.656^a^	-$7,125.56	$4,484.90
** Intercept**	7.383		0.000^a^		

*GLM* Generalized linear models, *USD* United States Dollar

^a^Wilcoxon rank sum test

**Table 6:** Determination of variance inflation factor (VIF)

**GLM 1**			**GLM 2**		
**Variable**	**VIF**	**1/VIF**	**Variable**	**VIF**	**1/VIF**
**Adverse events (0 = absent, 1 = present)**	1.470	0.682	**Adverse events (count predictor)**	2.000	0.500
**Hospitalization (0 = no, 1 = yes)**	1.550	0.644	**Hospitalization (0 = No, 1 = Yes)**	1.320	0.760
**Age (years)**	1.470	0.680	**Age (years)**	1.470	0.679
**Deaths**	1.440	0.696	**Deaths**	2.050	0.488
**Time to get ALL remission (days)**	1.280	0.782	**Time to get ALL remission (days)**	1.310	0.761
**Risk of neoplastic relapse (0 = standard, 1 = high)**	1.210	0.827	**Risk of neoplastic relapse (0 = standard, 1 = high)**	1.210	0.827
**Sex (0 = male, 1 = female)**	1.100	0.906	**Sex (0 = male, 1 = female)**	1.140	0.876
**Mean VIF**	1.360		**Mean VIF**	1.500	

This is the first study aiming to analyze the effect of ALL-related AEs on health care costs in pediatric population, so our results may help not only to local decision making but also it may contribute to the research agenda in this field.

## Discussion

As far as we know, this is the first study aiming to analyze the health care costs associated with AEs during induction remission chemotherapy in children with ALL, so our results may help not only to local decision making but also it may contribute to the research agenda in this field. At local level these results should alert both the health care providers that deliver oncologic treatment to pediatric patients with ALL as well as the government officers in charge of the program that grant financial support to the families to alleviate the burden of cancer, since according to our results the cost of AEs may be as large as the cost of chemotherapy.

We need to highlight the high frequency of AEs in the analyzed sample, since this fact not only has economic but also clinical and humanistic importance, for the deterioration in the quality of life of children during the RICP that is susceptible to economic estimation. This is a pending task in health economics, since the difficulties involved in assigning an economic value to suffering in children. We also could observe that although we recognized a high number of inpatient AEs in these patients, the target of ALL remission was not prevented in most of them. The percentage of remitted patients was comparable to remission rates observed in developed countries [1–4]. The patients who did not achieve ALL remission was because of early death during treatment. It has already been reported that in the care of complications for this group of patients in Mexico more than 50% of deaths occurred before the end of RICP [11]. According to the World Health organization (WHO), for pediatric population, all the AEs described above are common (> 1 in 100 people) [23].

Our study has some limitations. Being the medical records our main source of information to know the resources consumption, it was necessary to draw on some assumptions to derive the production function needed to micro-costing, since the medical notes are not designed to collect detailed economic data. As a result, even though the CTCAE favored identification of AEs according to SOC, unfortunately the data about resources not allowed us to estimate specific cost for each AE, so we restricted our costs calculations to an overall estimate of the cost of care in the presence of AEs. Additionally, coexistence of many AEs in the same patient represented a challenge to discriminate the different resources allocated to the specific treatment of each event and the clinical data necessary to classify AEs by severity were not available. These limitations however, lead to the planning of proper study designs like a matched case–control prospective aiming to estimate the attributable cost of AEs. Meanwhile, our results will help the hospital’s key stakeholders to allocate the budget efficiently, since these estimations were thought for financial decision-makers, not exactly for clinicians. Another apparent limitation concerning to the sample size, it was not entirely since we could evaluated some predictors of total cost using multivariate regression analysis.

It is not easy to contrast our results with other studies for several reasons. First, no costs estimates of AEs in pediatric patients during RICP has been published. Second, some non-recent studies have estimated, for example, the cost of infections in Nordic children with ALL ($18,747 per patient) [24], or the cost of severe complications in Chinese children with ALL ($20,932.71 per patient) [25], however the comparisons between our estimations and the mentioned are not direct since those health systems are very different from the Mexican one.

## Conclusions

The costs associated with AEs during RICP in Mexican children with ALL was $10,538.59 representing a high burden for the healthcare provider, that being one of the main pediatric oncological centers in the country, should take these figures into account when preparing budgets from public funds. The incremental cost of the treatment for a patient with AEs exceeded threefold the total cost of a patient without AEs. Generalized linear models showed that variables such as sex, risk category and alive status are associated with the total costs of AEs. This is the first study aiming to analyze the effect of ALL-related AEs on health care costs in pediatric population, so our results may help not only to local decision making but also it may contribute to the research agenda in this field.

## Supplementary Information

**Additional file 1.**

**Additional file 2.**

## Data Availability

The Raw Data is available in the supplementary file. If you required more information this is available from the corresponding author on reasonable request.

## Abbreviations

RICP: Remission-induction chemotherapy protocol
AEs: Adverse events
ALL: Acute Lymphoblastic Leukemia
SOC: System Organ Class
USD ($): United States Dollar
HIMFG: Hospital Infantil de México Federico Gómez
£: Pound sterling
UK: United Kingdom
CTCAE: Common Terminology Criteria for Adverse Events
GLM: Generalized linear models
